# Pragmatic adaptation of implementation research measures for a novel context and multiple professional roles: a factor analysis study

**DOI:** 10.1186/s12913-020-05118-4

**Published:** 2020-03-30

**Authors:** Justin D. Smith, Miriam R. Rafferty, Allen W. Heinemann, Mariah K. Meachum, Juan Villamar, Richard L. Lieber, C. Hendricks Brown

**Affiliations:** 1 Department of Psychiatry and Behavioral Sciences, Northwestern University Feinberg School of Medicine, Chicago, IL USA; 2 Department of Preventive Medicine, Northwestern University Feinberg School of Medicine, Chicago, IL USA; 3 Department of Pediatrics, Northwestern University Feinberg School of Medicine, Chicago, IL USA; 4 Department of Medical Social Sciences, Northwestern University Feinberg School of Medicine, Chicago, IL USA; 5 Center for Education in Health Sciences, Northwestern University Feinberg School of Medicine, Chicago, IL USA; 6 Department of Physical Medicine and Rehabilitation, Northwestern University Feinberg School of Medicine, Chicago, IL USA; 7 Shirley Ryan AbilityLab, Chicago, IL USA; 8 Departments of Physiology, Biomedical Engineering and Physical Medicine and Rehabilitation, Northwestern University, Chicago, IL USA

**Keywords:** Implementation, Adaptation, Pragmatic, Confirmatory factor analysis, Organizational change

## Abstract

**Background:**

Although some advances have been made in recent years, the lack of measures remains a major challenge in the field of implementation research. This results in frequent adaptation of implementation measures for different contexts—including different types of respondents or professional roles—than those for which they were originally developed and validated. The psychometric properties of these adapted measures are often not rigorously evaluated or reported. In this study, we examined the internal consistency, factor structure, and structural invariance of four well-validated measures of inner setting factors across four groups of respondents. The items in these measures were adapted as part of an evaluation of a large-scale organizational change in a rehabilitation hospital, which involved transitioning to a new building and a new model of patient care, facilitated by a significant redesign of patient care and research spaces.

**Methods:**

Items were tailored for the context and perspective of different respondent groups and shortened for pragmatism. Confirmatory factor analysis was then used to test study hypotheses related to fit, internal consistency, and invariance across groups.

**Results:**

The survey was administered to approximately 1208 employees; 785 responded (65% response rate) across the roles of clinician, researcher, leader, support staff, or dual clinician and researcher. For each of the four scales, confirmatory factor analysis demonstrated adequate fit that largely replicated the original measure. However, a few items loaded poorly and were removed from the final models. Internal consistencies of the final scales were acceptable. For scales that were administered to multiple professional roles, factor structures were not statistically different across groups, indicating structural invariance.

**Conclusions:**

The four inner setting measures were robust for use in this new context and across the multiple stakeholder groups surveyed. Shortening these measures did not significantly impair their measurement properties; however, as this study was cross sectional, future studies are required to evaluate the predictive validity and test-retest reliability of these measures. The successful use of adapted measures across contexts, across and between respondent groups, and with fewer items is encouraging, given the current emphasis on designing pragmatic implementation measures.

## Background

Measurement issues in dissemination and implementation research are some of the most pressing challenges in the field due to a lack of psychometric studies and a tendency for scales to need adaptation for specific contexts and respondents [1, 2]. As implementation research expands within the field of health services research, it is important that healthcare researchers are aware of best practices in implementation measurement [2]. Several implementation measures have been developed and validated in the social sciences and mental health fields to assess, for example, attitudes toward use of evidence-based practices (EBPs), organizational culture, and implementation leadership [3–5]. Using validated measures, or providing psychometric data when using existing measures in a new context, will improve the reproducibility and comparability of research on implementation determinants and processes [1]. Further, given the overall dearth of well-established measures for specific contexts and stakeholder types, researchers often adapt existing measures to align with the aims of a new study. Not only is this common practice, it is specifically endorsed as a research priority in the recent re-issue of the National Institutes of Health funding opportunity announcements on Dissemination and Implementation Research in Health posted May 8, 2019 (https://grants.nih.gov/grants/guide/pa-files/PAR-19-274.html), in which researchers are discouraged from developing new measures solely for use in a specific study and are instead encouraged to use standard, validated measures where possible.

However, adapting a measure for a new context may affect the reliability and validity of the measure, necessitating re-evaluation of the measurement properties. We cannot assume that the construct being measured is salient across contexts as diverse as elementary schools, mental healthcare, or hospital-based adult medicine, because respondents may interpret items differently. Adaptations may include word changes to improve the specificity and interpretability of items for the new context and new participants, respectively. However, the psychometric properties of few implementation research measures have been formally evaluated across diverse health care contexts.

There is also a growing emphasis on pragmatic measures in implementation research. Among other characteristics, pragmatic measures should be salient to both the stakeholders and researchers, be perceived as low burden, and have broad applicability [6–8]. However, adapting validated measures to make them more pragmatic (i.e.*,* more salient, shorter, and with broader application) could result in reducing the psychometric stability of the original measure. Thus, when adapting established implementation research questionnaires for a new context, it is imperative to verify that properties such as internal consistency and factor structure are acceptable and comparable to the original measure. For example, the Implementation Leadership Scale (ILS), was originally validated in social services settings and subsequently applied to substance use disorder treatment organizations. Aarons et al. [3] found that the properties of the ILS in this new context were acceptable and consistent with the original measure.

It is also important to understand whether internal consistency and factor structure of validated scales are adequate across different stakeholder roles, because inherent differences in perspective could translate to a different interpretation of the items. Current implementation research models and frameworks specify that obtaining data from people with varied perspectives is necessary [1, 9]. However, stakeholders with different professional roles within complex health systems may have different perspectives on the implementation of innovations [10, 11]. These differences may reflect actual differences in perspective or simply differences in the questionnaire’s constructs between groups that is unintended or unanticipated. It is therefore important to look beyond simple mean-level differences to determine whether the psychometric properties of the questionnaire vary between stakeholder groups. It may also be beneficial to assess structural variance within confirmatory factor analysis to test for differences between respondent groups.

The purpose of this report is to describe our process for adapting validated implementation research measures to reflect the context and the distinct perspectives of key stakeholder groups in a large organizational change that involved changes in physical space and leadership structure, as well as changes in personnel and team roles. Specifically, the change was the transition of a major urban academic hospital, the Rehabilitation Institute of Chicago (RIC) to a new building, named the Shirley Ryan AbilityLab (SRAlab), and a new model of patient care that emphasizes collaborative efforts among clinicians and investigators. Prior to this transition, we were interested in examining the (1) internal consistency, (2) factor structure, and (3) variation in factor structures in implementation research measures across stakeholder groups such as clinicians, researchers, leaders, support staff, or dual-role clinician-researchers. Variation in factor structure was important due to differing item sets in some cases. In addition, our observations during transition preparations led us to hypothesize that the interpretation and understanding of items may differ based on stakeholders’ roles within the organization.

## Methods

### Context

In 2015, a group of implementation researchers from Northwestern University and researchers from the SRALab, an academic physical medicine and rehabilitation hospital, began a partnership to evaluate an upcoming organizational change. The patients, clinical staff, and research scientists of the RIC were relocating to the SRAlab, a newly constructed building one block away. This transition involved a physical move to a state-of-the-art building, a reorganization of leadership, and a significant change in patient care and research practices: patient-care floors were designed to accommodate research staff alongside healthcare providers, with the goal of increasing collaboration between researchers and clinicians.

The clinical-academic team developed a survey to evaluate the implementation factors that would contribute to, and were affected by, the transition to the new model of care, which included (1) all private rooms, (2) improved technology integration, (3) research labs embedded within clinical therapy spaces, and consequently (4) increased interactions between clinicians and researchers.

The survey was administered 3 months prior to the transition, with plans to re-administer the survey periodically in the years following the transition.

### Procedures

#### Measure selection

We initially identified four validated implementation research measures to evaluate (1) leadership climate, (2) beliefs about the upcoming transition, and individuals’ (3) use of and (4) attitudes toward EBPs. Consistent with the Consolidated Framework for Implementation Research (CFIR) [12], the selected measures assessed determinants in the domains of “characteristics of the intervention”, or model of care, and the “inner setting”, which were hypothesized to be the most relevant to the transition. These constructs have been shown to impact the uptake of innovations in healthcare delivery systems [13].

#### Measure adaptation

We adapted each measure to the SRAlab context and the professional roles we intended to survey. Instrument adaptations were discussed by a workgroup comprised of investigators with expertise in implementation research, organizational and systems change, and rehabilitative services provided by RIC, together with representatives from the three primary roles in RIC that we intended to survey: clinical staff, researchers, and leaders (dual-role leader/researcher). The workgroup selected subscales and items from each measure that were deemed salient to the transition and tailored each selected item for the various stakeholder groups, to ensure relevance. For example, in the Implementation Leadership Scale [14], we adapted the question originally developed by Aarons et al. “[Name of Supervisor] supports employee efforts to learn more about evidence-based practice” to “RIC’s leadership team supports clinicians’ efforts to learn more about research” (to be asked of clinicians) and to “RIC’s leadership team supports researchers’ efforts to use clinical practice to drive research development” (to be asked of researchers). In order to be more pragmatic, we removed less relevant or redundant items and subscales through a series of meetings with leadership and pilot testing with representatives from each of the professional roles. The feedback was used to refine the remaining items. A list of the adapted items for each role is presented in Table 1.

**Table 1:** Items from original surveys included in our study, and adapted wording for each self-reported primary role

Scale/Item	Original Item wording	Role-Specific Adapted Wording			
		Clinician	Researcher	Leadership	Support Staff
*Implementation Leadership Scale (ILS)*	*Please indicate the extent to which you agree with each statement.*	*Please indicate the extent to which you agree with each statement about the leadership team’s role in RIC’s transition to the AbilityLab Model of Care using the following scale.* *1: Not at All; 2: To a Slight Extent; 3: To a Moderate Extent; 4: To a Great Extent; 5: To a Very Great Extent*			
lsplrnA	[Name of Supervisor] supports employee efforts to learn more about evidence-based practice.	RIC’s leadership team supports clinicians’ efforts to learn about research.	RIC’s leadership team supports researchers’ efforts to learn about clinical practice.	Average of “RIC’s leadership team supports clinicians’ efforts to learn about research” & “RIC’s leadership team supports researchers’ efforts to learn about clinical practice.”	N/A
lspebpA	[Name of Supervisor] supports employee efforts to use evidence-based practice.	RIC’s leadership team supports clinicians’ efforts to use research in clinical practice.	RIC’s leadership team supports researchers’ efforts to use clinical practice to drive research development.	Average of “RIC’s leadership team supports researchers’ efforts to use clinical practice to drive research development.” & “RIC’s leadership team supports clinicians’ efforts to use research in clinical practice.”	RIC’s leadership team supports employees’ efforts to use research to inform their work.
lapprec	[Name of Supervisor] recognizes and appreciates employee efforts toward successful implementation of evidence-based practice.	RIC’s leadership team recognizes and appreciates employee efforts toward successful implementation of the AbilityLab Model of Care.	RIC’s leadership team recognizes and appreciates employee efforts toward successful implementation of the AbilityLab Model of Care.	RIC’s leadership team recognizes and appreciates employee efforts toward successful implementation of the AbilityLab Model of Care.	RIC’s leadership team recognizes and appreciates employee efforts toward successful implementation of the AbilityLab Model of Care.
lremobst	[Name of Supervisor] has removed obstacles to the implementation of evidence-based practice.	RIC’s leadership team has removed obstacles to implementing the AbilityLab Model of Care.	RIC’s leadership team has removed obstacles to implementing the AbilityLab Model of Care.	RIC’s leadership team has removed obstacles to implementing the AbilityLab Model of Care.	RIC’s leadership team has removed obstacles to implementing the AbilityLab Model of Care.
lanswerq	[Name of Supervisor] is able to answer my questions about evidence-based practice.	My direct supervisor is able to answer my questions about the AbilityLab Model of Care.	My direct supervisor is able to answer my questions about the AbilityLab Model of Care.	My direct supervisor is able to answer my questions about the AbilityLab Model of Care.	My direct supervisor is able to answer my questions about the AbilityLab Model of Care.
lopenprb	[Name of Supervisor] reacts to critical issues regarding the implementation of evidence-based practice by openly and effectively addressing the problem(s).	My direct supervisor openly addresses problems regarding the implementation of new processes.	My direct supervisor openly addresses problems regarding the implementation of new processes.	My direct supervisor openly addresses problems regarding the implementation of new processes.	My direct supervisor openly addresses problems regarding the implementation of new processes.
*Organizational Change Recipients Beliefs Scale (OCRBS)*	A seven-cell format with the anchors being strongly disagree and strongly agree.	Please indicate the extent to which you agree with each statement about RIC’s transition to the AbilityLab Model of Care using the following scale. 1: Strongly Disagree; 2: Disagree; 3: Neutral; 4: Agree; 5: Strongly Agree			
wneed	We need to improve the way we operate in this organization.	We need to improve the way we deliver care at RIC.	We need to improve the way we deliver care at RIC.	We need to improve the way we deliver care at RIC.	We need to improve the way we deliver care at RIC.
wcanimpl	We have the capability to successfully implement this change.	We have the capability to successfully implement the AbilityLab Model of Care.	We have the capability to successfully implement the AbilityLab Model of Care.	We have the capability to successfully implement the AbilityLab Model of Care.	We have the capability to successfully implement the AbilityLab Model of Care.
icanimpl	I can implement this change in my job.	I can implement the AbilityLab Model of Care.	I can implement the AbilityLab Model of Care.	I can implement the AbilityLab Model of Care.	I can implement the AbilityLab Model of Care.
goodpt	I believe the proposed organizational change will have a favorable effect on our operations.	Patients will benefit from the change from RIC’s current model of rehabilitation to the AbilityLab Model of Care.	Patients will benefit from the change from RIC’s current model of rehabilitation to the AbilityLab Model of Care.	Patients will benefit from the change from RIC’s current model of rehabilitation to the AbilityLab Model of Care.	Patients will benefit from the change from RIC’s current model of rehabilitation to the AbilityLab Model of Care.
goodme	This change will benefit me.	I will benefit from the change from RIC’s current model of rehabilitation to the AbilityLab Model of Care.	I will benefit from the change from RIC’s current model of rehabilitation to the AbilityLab Model of Care.	I will benefit from the change from RIC’s current model of rehabilitation to the AbilityLab Model of Care.	I will benefit from the change from RIC’s current model of rehabilitation to the AbilityLab Model of Care.
iprepare	I am capable of successfully performing my job duties with the proposed organizational change.	I am prepared to be a part of the AbilityLab Model of Care.	I am prepared to be a part of the AbilityLab Model of Care.	I am prepared to be a part of the AbilityLab Model of Care.	I am prepared to be a part of the AbilityLab Model of Care.
ifulfill	With this change in my job, I will experience more self-fulfillment.	I will experience more self-fulfillment with the AbilityLab Model of Care.	I will experience more self-fulfillment with the AbilityLab Model of Care.	I will experience more self-fulfillment with the AbilityLab Model of Care.	I will experience more self-fulfillment with the AbilityLab Model of Care.
peerembr	Most of my respected peers embrace the proposed organizational change.	Most of my peers have embraced the AbilityLab Model of Care.	Most of my peers have embraced the AbilityLab Model of Care.	Most of my peers have embraced the AbilityLab Model of Care.	Most of my peers have embraced the AbilityLab Model of Care.
*Evidence Based Practice Questionnaire (EBPQ)* Practice Subscale	Considering your practice in relation to an individual patient’s care over the *past* year, how often have you done the following in response to a gap in your knowledge.	Considering a gap in your knowledge related to a patient’s care in the past 2 months, how often have you done the following? Note: Choose n/a if you have not had gaps in your knowledge regarding patient care in the past 2 months. 1: Never; 2: Rarely; 3: Occasionally; 4: Often; 5: Very Often; 6: n/a (option n/a set to missing in analyses)			
quesplaq	Formulated a clearly answerable question as the beginning of the process towards filling this gap:	Seriously questioned whether your default plan of care was the best option.	N/A	N/A	N/A
litsrchq	Tracked down the relevant evidence once you have formulated the question:	Searched the literature to answer a question related to alternative plans of care.	N/A	N/A	N/A
integreq	Integrated the evidence you have found with your expertise:	Integrated the evidence you found in the literature with your plan of care.	N/A	N/A	N/A
evaleffq	Evaluated the outcomes of your practice:	Evaluated the patient’s outcomes to assess if your plan of care was effective.	N/A	N/A	N/A
sharepbq	Shared this information with colleagues:	Shared your practice-based evidence with colleagues.	N/A	N/A	N/A
*Evidence-Based Practice Attitudes Scale (EBPAS)* Openness Subscale [1]	The following questions ask about your feelings about using new types of therapy, interventions, or treatments. Manualized therapy, treatment, or intervention refers to any intervention that has specific guidelines and/or components that are outlined in a manual and/or that are to be followed in a structured or predetermined way. Indicate the extent to which you agree with each item.	Please indicate the extent to which you agree with each statement about using new techniques or outcome measures with the following scale. 1: Not at All; 2: To a Slight Extent; 3: To a Moderate Extent; 4: To a Great Extent; 5: To a Very Great Extent			
ilikenew [1]	I like to use new types of therapy/interventions to help my clients.	I like to use new techniques or outcome measures to help my patients.	N/A	N/A	N/A
eagnewCR [1]	I am willing to use new and different types of therapy/interventions developed by researchers.	I am eager to use new and different techniques or outcome measures developed by researchers.	I would be eager to do research in a new area if a clinician thought it was important.	N/A	N/A
trydiff [1]	I would try a new therapy/intervention even if it were very different from what I am used to doing.	I would try new techniques or outcome measures even if it were very different from what I am used to doing.	N/A	N/A	N/A
*Evidence-Based Practice Attitudes Scale (EBPAS)* Other questions [2]	If you received training in a therapy or intervention that was new to you, how likely would you be to adopt it if …	If you have received or were to receive training in a technique or outcome measure that was new to you, how likely would you be to adopt it if ... 1: Not at All; 2: To a Slight Extent; 3: To a Moderate Extent; 4: To a Great Extent; 5: To a Very Great Extent			
intuitive [2]	it was intuitively appealing?	It was intuitively appealing?	N/A	N/A	N/A
reqbysup [2]	it was required by your supervisor?	It was required by your supervisor?	N/A	N/A	N/A
colhappy [2]	it was being used by colleagues who were happy with it?	It was being used by colleagues who were happy with it?	N/A	N/A	N/A
iknomoCRz [2]	I know better than academic researchers how to care for my clients. (reverse scored)	I know better than academic researchers how to care for my patients. (reverse scored)	I know better than clinicians how new techniques or measures in my area of research could improve patient care. (reverse scored)	N/A	N/A

#### Survey administration

After receiving IRB approval from Northwestern University, the survey was administered using Research Electronic Data Capture (REDCap), a web-based application for data collection and management, hosted at Northwestern University Feinberg School of Medicine [15, 16]. Active email addresses (*n* = 1208) in the human resources system—the best means of contacting all current employees—were used to invite employees to take the survey, via email, over 7 weeks, from 1/17/2017 to 3/3/2017, beginning 3 months before the transition. Email communication via REDCAP ensured participant anonymity while permitting follow up of nonrespondents. We used several strategies to encourage participation, including offering incentives (a customized mug with the SRAlab logo, a raffled dinner with the RIC Chief Executive Officer), periodic electronic mail prompts, and in-person reminders during clinical and research team meetings. Written informed consent was obtained electronically prior to administration of the online survey.

### Participants

Question banks were adapted for and administered based on four self-reported primary roles: clinician (physicians, nurses, and allied health therapists), researcher, leader, or support staff. Secondary roles were also reported, allowing us to generate five analysis categories accounting for dual roles: clinician only, researcher only, support staff only, leaders (including those with primary and secondary leadership roles), and people with dual roles as a clinician/researcher. A total of 785 employees completed the survey, for an overall response rate of 65%. Response rates by primary role were 63% for clinicians (*n* = 544), 58% for researchers (*n* = 100), 92% for leaders (*n* = 79), and 64% for support staff (*n* = 52). Response rates were approximate, as we were unable to verify that all of the 1208 unique email addresses were active. For example, the 43 surveys that were returned as undeliverable, suggesting that the person was no longer employed at RIC or that they used a different address, were included in the total number of surveys administered. Respondents were predominantly female (77%), White (76%) (Black: 13%; Asian: 10%; other race/ethnicity< 1%), and most had been employed in the hospital for less than 10 years (73%), with 53% reporting less than 5 years’ employment.

### Measures

***Implementation Leadership Scale (ILS)*** comprises 12 items that are rated on a 5-point scale indicating the degree to which the leader performs a specific behavior [14]. The 4 original subscales include *Proactive Leadership* (4 items), *Knowledgeable Leadership* (4 items), *Supportive Leadership* (4 items), and *Perseverant Leadership* (4 items). The mean of the subscales is computed to create the ILS total mean score (α = 0.97). Internal consistencies of the original subscales and total ILS score range from α = 0.93–0.98 in published studies [3, 14]. Our adapted ILS included 6 items: 1 from the Proactive subscale, 1 from Knowledgeable subscale, 3 from Supportive subscale, and 1 from the Perseverant subscale. All professional roles except for support staff answered these questions regarding the leadership team, including leaders. For this and all other measures, subscale scores were only computed when at least three items from the original subscale were used (see Data Analysis section).

***Organizational Change Recipients’ Beliefs Scale (OCRBS)*** is a 24-item scale to assess respondents’ beliefs about a current or proposed change, to gauge the degree of buy-in among recipients and assess beliefs about *Discrepancy* (4 items), *Appropriateness* (5 items), *Efficacy* (4 items), *Principal Support* (6 items), and V*alence* (4 items) that could adversely impact the success of the change [4]. Internal consistencies of the original scales ranged from α = 0.86–0.95 in the original validation [4]. In our study, all participants answered 8 items: 1 in Discrepancy, 1 in Appropriateness, 3 in Efficacy, 1 in Principal Support, and 2 in Valance. We modified the original 7-cell anchored format to a 5-point Likert scale to be consistent with our other items. This change to a 5-point scale resulted from our bench testing procedures, during which time feedback was given that it was clearer and easier to interpret differences between points on the 5-piont scales than it was for the instruments with 7-point scales. This change was also made to the Evidence-Based Practice Questionnaire for the same reasons.

***Evidence-Based Practice Questionnaire (EBPQ)*** comprises 24 items and was developed to measure nurse’s EBP use, attitudes, and knowledge [17]. Internal consistencies are acceptable with Cronbach’s alpha (α) of 0.87 for the full questionnaire; α = 0.85 for the *Practice of EBP* subscale; α = 0.79 for the *Attitude towards EBP* subscale; and α = 0.91 for the *Knowledge/Skills associated with EBP* subscale [17]. Our adapted survey included 5 items from the Practice subscale for clinicians and a change to a 5-point scale.

***Evidence-Based Practice Attitudes Scale (EBPAS)*** comprises 15 items across four dimensions pertaining to attitudes of mental health therapists toward adopting and delivering EBPs: *openness* to new practices (4 items); intuitive *appeal* of EBP (4 items); likelihood of adopting EBP given the *requirements* to do so (3 items); and perceived *divergence* of usual practices from research-based, academically-developed interventions (4 items) [5]. Internal consistency of the original subscales in the original study of the EBPAS ranged from α = 0.59 to α = 0.90 with an overall α = 0.77 [5]. Our adapted survey included 7 items for clinicians (3 from Openness, 2 from Appeal, 1 from Requirements, and 1 from Divergence).

### Data analysis

Data analyses were conducted in Mplus 8 [18] using maximum likelihood estimation to conduct a confirmatory factor analysis (CFA), while correlations, internal consistency, and descriptive statistics were completed in SAS 9.4. Determination of model fit included standard indicators: comparative fit index (CFI) [19], the root mean square error of approximation (RMSEA) [20], and the weighted root mean residual (WRMR) [21]. Good fit to the data was indicated by CFI values greater than 0.93 [22], RMSEA values less than 0.06, and WRMR values less than 1.0 [18, 23, 24]. First, we conducted an independent CFA for each measure by replicating the original subscales when at least 3 items were available, or by including all items in a single overall scale when subscales could not be specified. We removed items when there was evidence of low contribution to the underlying construct, as evidenced by standardized factor loadings < 0.5, and allowed items within scales/subscales to correlate when doing so resulted in a significant improvement in model fit. We tested for structural invariance by professional role for the ILS and OCRBS after fitting an acceptable overall CFA model using a Wald Test, to determine whether role of the respondent was related to differences in the factor loadings of the latent variables. Variances and standard errors were allowed to vary across roles. We report omnibus tests of mean differences in each scale by professional role.

## Results

Table 2 shows internal consistency and final CFA models for each scale. Two items were eliminated from the OCRBS model, one from the EBPAS, and one from the EBPQ. Each final model provided acceptable fit to the data and the standardized factor loadings were statistically significant (*p* < 0.001), ranging from 0.63 to 0.95 for the retained items, indicating that the factors, with correlated items when appropriate, contributed to the latent construct. The multiple-group CFA by professional role approached statistical significance on the ILS (Wald [10] =16.71, *p* = 0.08) and OCRBS (Wald [11] = 19.33, *p* = 0.06). Supplemental Tables S1, S2, S3, S4 report the inter-correlations and descriptive statistics for each scale. To summarize the results in the Supplemental Tables, the final included items were significantly and strongly intercorrelated within measures, with a range of Spearman’s *r* = .48–.84 on the ILS (Table S1), *r* = .41–.69 on the OCRBS (Table S2), *r* = .32–.73 on the EBPQ (Table S3), and *r* = .42–.83 on the EBPAS (Table S4).

**Table 2:** Factor loadings, fit statistics, and correlations between items for the final model results

	Fit Statistics							Parameter Estimates (factor loadings, correlations)			
Scale/Subscale/Item	χ^2^(df)	*p*	CFI	RMSEA	90% CI	WRMR	α	*B*	*SE (B)*	β	95% CI
ILS	7.523(5)	.185	1.00	.03	.000 | .060	.17	.89				
*Factor Loadings*											
RIC’s leadership team supports clinicians’ efforts to learn about research.								1.00***	.00	.81	.786 | .842
RIC’s leadership team supports clinicians’ efforts to use research in clinical practice.								.97***	.04	.77	.744 | .804
RIC’s leadership team recognizes and appreciates employee efforts toward successful implementation of the AbilityLab Model of Care.								2.64***	.22	.91	.890 | .935
RIC’s leadership team has removed obstacles to implementing the AbilityLab Model of Care.								1.94***	.14	.85	.825 | .881
My direct supervisor is able to answer my questions about the AbilityLab Model of Care.								1.09***	.07	.68	.637 | .714
My direct supervisor openly addresses problems regarding the implementation of new processes.								1.04***	.07	.66	.612 | .706
*Correlations between items (WITH statements)*											
My direct supervisor openly addresses problems regarding the implementation of new processes. WITH My direct supervisor is able to answer my questions about the AbilityLab Model of Care.								.55***	.03	.55	.496 | .594
My direct supervisor openly addresses problems regarding the implementation of new processes. WITH RIC’s leadership team has removed obstacles to implementing the AbilityLab Model of Care.								−.30***	.06	−.30	−.412 | -.197
My direct supervisor openly addresses problems regarding the implementation of new processes. WITH RIC’s leadership team recognizes and appreciates employee efforts toward successful implementation of the AbilityLab Model of Care.								−.22***	.07	−.22	−.353 | -.092
RIC’s leadership team supports clinicians’ efforts to use research in clinical practice. WITH RIC’s leadership team supports clinicians’ efforts to learn about research.								.23***	.02	.58	.537 | .614
Item(s) removed during CFA: None											
OCRBS	2.785(4)	.595	1.00	.00	.000 | .045	.16	.88				
*Factor Loadings*											
I will experience more self-fulfillment with the AbilityLab Model of Care.								1.00***	.00	.86	.827 | .892
I will benefit from the change from RIC’s current model of rehabilitation to the AbilityLab Model of Care.								1.17***	.09	.90	.860 | .923
Most of my peers have embraced the AbilityLab Model of Care.								.64***	.06	.74	.696 | .774
We have the capability to successfully implement the AbilityLab Model of Care.								.60***	.05	.71	.669 | .750
Patients will benefit from the change from RIC’s current model of rehabilitation to the AbilityLab Model of Care.								.82***	.07	.81	.782 | .838
I can implement the AbilityLab Model of Care.								.56***	.05	.69	.644 | .727
*Correlations between items (WITH statements)*											
I will experience more self-fulfillment with the AbilityLab Model of Care. WITH I will benefit from the change from RIC’s current model of rehabilitation to the AbilityLab Model of Care.								.28***	.08	.28	.119 | .439
I will experience more self-fulfillment with the AbilityLab Model of Care. WITH I can implement the AbilityLab Model of Care.								.17***	.04	.17	.083 | .246
Patients will benefit from the change from RIC’s current model of rehabilitation to the AbilityLab Model of Care. WITH We have the capability to successfully implement the AbilityLab Model of Care.								.35***	.04	.35	.270 | .427
I can implement the AbilityLab Model of Care. WITH We have the capability to successfully implement the AbilityLab Model of Care.								.20***	.04	.20	.125 | .280
Most of my peers have embraced the AbilityLab Model of Care. WITH I will benefit from the change from RIC’s current model of rehabilitation to the AbilityLab Model of Care.								−.25***	.06	−.25	−.374 | -.128
Item(s) removed during CFA: We need to improve the way we deliver care at RIC; I am prepared to be a part of the AbilityLab Model of Care.											
EBPQ (clinicians only)	.000 (0)	.000	1.00	.00	.00 | .00	.00	.82				
*Factor Loadings*											
Searched the literature to answer a question related to alternative plans of care.								1.00***	.00	.85	.805 | .891
Evaluated the patient’s outcomes to assess if your plan of care was effective.								.50***	.05	.63	.560 | .692
Shared your practice-based evidence with colleagues.								.56***	.05	.67	.608 | .722
Integrated the evidence you found in the literature with your plan of care.								1.68***	.44	.94	.895 | .980
*Correlations between items (WITH statements)*											
Shared your practice-based evidence with colleagues. WITH Evaluated the patient’s outcomes to assess if your plan of care was effective.								.32***	.05	.32	.229 | .418
Evaluated the patient’s outcomes to assess if your plan of care was effective. WITH Searched the literature to answer a question related to alternative plans of care.								−.34***	.09	−.34	−.507 | -.163
Item(s) removed during CFA: Seriously questioned whether your default plan of care was the best option.											
EBPAS (clinicians only)	7.256(6)	.298	1.00	.02	.000 | .064	.22					
Factor 1: Openness							.90				
*Factor Loadings*											
I am eager to use new and different techniques or outcome measures developed by researchers.								1.00***	.00	.89	.854 | .925
I would try new techniques or outcome measures even if it were very different from what I am used to doing.								.89***	.04	.95	.919 | .976
I like to use new techniques or outcome measures to help my patients.								1.02***	.04	.90	.862 | .947
*Correlations between items (WITH statements)*											
I like to use new techniques or outcome measures to help my patients. WITH I would try new techniques or outcome measures even if it were very different from what I am used to doing.								−.07***	.02	−.59	−1.138 | -.047
Factor 2: Appeal/Requirement							.82				
*Factor Loadings*											
it was being used by colleagues who were happy with it?								1.00	.00	.88	.838 | .915
it was intuitively appealing?								1.02***	.04	.90	.864 | .930
it was required by your supervisor?								.88***	.04	.77	.720 | .821
*Correlations between items (WITH statements)*											
it was required by your supervisor? WITH it was intuitively appealing?								−.08***	.03	−.30	−.503 | -.075
Correlation between Factors Open WITH Appeal/Requirement								.54***	.03	.70	.645 | .745
Item(s) removed during CFA: I know better than academic researchers how to care for my clients (reverse scored) (did not fit in either subscale)											

*Note*. ^***^p < .001. *B* = unstandardized factor loadings. β = standardized factor loadings. *EBPQ* Evidence Based Practice Questionnaire, *EBPAS* Evidence Based Practice Attitudes Scale, *ILS* Implementation Leadership Scale. *OCBRS* Organizational Change Recipient’s Belief Scale. Item key in Table 1. Only correlations between items and factors specified in the final model are reported; complete intercorrelations available in Supplemental Tables S1, S2, S3, S4

Mean values of the scales or subscales were calculated based on the factors included in the final CFA models. A significant difference between professional roles was found in the mean ILS (*F* [3, 721] = 6.27, *p* < 0.001), such that leaders rated leadership support higher than did researchers and dual-role clinician/researchers. Clinicians also rated their leaders higher on the ILS than did the dual-role clinician/researchers. In addition, there was a significant difference in mean OCRBS between roles (*F* [4, 787] = 5.38, *p* < 0.001), such that researchers reported less buy-in to the proposed change to the SRAlab and the new model of care compared to clinicians, support staff, and leaders.

## Discussion

The assumption that the properties of an implementation measure construct will hold when administered in different contexts and to various stakeholders is rarely tested and applying measures without appropriate customization could lead to misinterpretation. Our results indicated that measures of leadership climate, beliefs regarding change, and use of and attitudes toward EBP had adequate internal consistency and factor loadings. However, in some cases, the best fitting CFA model required removing additional items—further shortening the original scale. This could have been in part due to inclusion of single items from the original subscales that, when combined with the remaining items, resulted in poor factor loadings on a general construct. Although the measures of leadership support and beliefs about change did not differ significantly based on the respondent’s role, the data suggest that caution is required when applying brief implementation measures to people with different roles in an organization.

These findings are promising for several reasons. First, they demonstrate the robustness of four common implementation research measures used to assess the inner setting subdomains of leadership climate, beliefs regarding change, and use of and attitudes toward EBPs, even when these measures are shortened for pragmatism and adapted.

Second, our results indicate that tailoring the items of well-validated scales to new contexts and for specific stakeholder perspectives is feasible and empirically supported. While the current structure of the ILS guides researchers to tailor the question stems for a specific context, our results support considering this approach during the development and validation of new pragmatic measures. Tailoring items could result in better predictive validity of these measures by reducing error variance and misinterpretation of general items applied to a specific problem or viewpoint. In most implementation research studies, some adaptation of items to the context of the study is necessary or preferable to obtain valid and reliable results. This study also shows that some measures developed for a specific context might contain items that do not translate well to other contexts. This is exemplified by removal of some items during CFA, even after the items had been selected as relevant and tailored to better match this specific context by key stakeholders.

Third, results show that shortened versions of some implementation research measures can be developed. However, the shortened measures used in this study resulted in only a single item from some of the original subscales, which reduces specificity for addressing research questions that require a psychometrically robust scale. Although we did not test the predictive validity of the adapted scales, establishing that the measure has adequate internal consistency, factor loadings, and is invariant across respondent groups, is a necessary first step.

Additional File 1 includes the adapted versions of the ILS, OCRBS, EBPQ, and EBPAS resulting from this study. Scoring for each adapted scale simply involves calculating the mean.

### Limitations

Limitations of this study include the potential loss of specificity due to the shortening of measures. For example, the original EPBAS had four subscales. Our final model replicated the openness subscale and created a new subscale containing items from both the appeal and requirement subscales of the original measure that relate to the likelihood to use EBP. Were our research questions and hypotheses specific to the independent role of each of these subscales, it would not have been advisable to reduce the items. Future research could address the reliability, construct, and predictive validity of our adapted measures [25]. Additionally, future work could include analyses based on item response theory, rather than using CFA, to determine the appropriateness of our reduced scales and removal of items. In this study, we confirmed our CFA results by documenting internal consistency with and without the dropped item(s). A second limitation is the reliance on one healthcare organization. Future research to replicate these findings across organization types and with different respondents is needed. However, this study supports continued evaluation of this specific organizational change with confidence in our measurement approach. Last, this study includes only a handful of the subdomains of the CFIR, which has measures for many but not all of the subdomains. The Society for Implementation Research Collaboration Instrument Review Project has compiled a comprehensive repository of available measures for each subdomain [6, 26]. Although generalizability of each measure was not included in their review and ratings, even a cursory scan of the included measures suggests that some are quite specific to a particular service context, respondent, or clinical practice. These measures could prove more challenging to adapt than the more general measures described in this paper.

## Conclusions

This study demonstrates methods for adapting and shortening implementation research measures and examining the impact on multiple psychometric properties. We selected measures that are widely used and whose original versions are psychometrically sound. However, evaluation studies are needed for other implementation measures. Similarly, development of new measures should include their evaluation in diverse contexts and with varied stakeholders. With a current emphasis on more pragmatic implementation research measures [8], these results are encouraging from the standpoint of use across contexts, with different respondent groups, and with reduced item counts. Validating adaptations of existing measures and publication of cross-informant and cross-setting psychometric evaluations such as this can help to address the noted gaps and shortcomings of implementation research instrumentation.

## Supplementary information

**Additional file 1: Table S1.** Intercorrelations and descriptive statistics for ILS. **Table S2.** Intercorrelations and descriptive statistics for OCRBS. **Table S3.** Intercorrelations and descriptive statistics for EBPQ. **Table S4.** Intercorrelations and descriptive statistics for EBPAS.

**Additional file 2: Supplemental File 1.** Adapted Versions of the Scales

## Data Availability

Data is available upon request to the corresponding author.

## Abbreviations

EBP: Evidence-based practices
ILS: Implementation leadership scale
RIC: Rehabilitation institute of Chicago
SRAlab: Shirley Ryan abilitylab
CFIR: Consolidated framework for implementation research
EBPQ: Evidence-based practice questionnaire
EBPAS: Evidence-based practice attitudes scale
OCRBS: Organizational change recipients’ beliefs scale
CFA: Confirmatory factor analysis
CFI: Comparative fit index
RMSEA: Root mean square error of approximation
WRMR: Weighted root mean residual
