# Supplementary material for: Pragmatic adaptation of implementation research measures for a novel context and multiple professional roles: a factor analysis study
Source: BMC Health Serv Res. 2020 Mar 30;20:257. doi: 10.1186/s12913-020-05118-4 (PMC7106795; doi:10.1186/s12913-020-05118-4)
Supplement: Supplementary file 1 — Additional file 1: Table S1. Intercorrelations and descriptive statistics for ILS. Table S2. Intercorrelations and descriptive statistics for OCRBS. Table S3. Intercorrelations and descriptive statistics for EBPQ. Table S4. Intercorrelations and descriptive statistics for EBPAS. [file 12913_2020_5118_MOESM1_ESM.docx]

**SUPPLEMENTAL TABLES 1–4**

Table S1. *Intercorrelations and descriptive statistics for ILS*

| Variable | 1 | 2 | 3 | 4 | 5 | 6 |
| --- | --- | --- | --- | --- | --- | --- |
| 1. RIC's leadership team supports clinicians' efforts to learn about research. | – | .84*** | .73*** | .65*** | .51*** | .52*** |
| 1. RIC's leadership team supports clinicians' efforts to use research in clinical practice. |  | – | .69*** | .60*** | .50*** | .51*** |
| 1. RIC's leadership team recognizes and appreciates employee efforts toward successful implementation of the AbilityLab Model of Care. |  |  | – | .69*** | .55*** | .48*** |
| 1. RIC's leadership team has removed obstacles to implementing the AbilityLab Model of Care. |  |  |  | – | .52*** | .40*** |
| 1. My direct supervisor is able to answer my questions about the AbilityLab Model of Care. |  |  |  |  | – | .69*** |
| 1. My direct supervisor openly addresses problems regarding the implementation of new processes. |  |  |  |  |  | – |
| Mean | 3.23 | 3.33 | 3.13 | 2.90 | 3.36 | 3.45 |
| Standard deviation | 1.04 | 1.05 | 1.06 | .92 | .98 | 1.10 |
| N | 742 | 739 | 740 | 732 | 736 | 734 |
| *Note*. ^*^*p* < .05. ^**^*p* < .01. ^***^*p* < .001 (two-tailed). Correlations are in Spearman’s *r*. | | | | | | |

Table S2. *Intercorrelations and descriptive statistics for OCRBS*

| Variable | 1 | 2 | 3 | 4 | 5 | 6 | 7 | 8 |
| --- | --- | --- | --- | --- | --- | --- | --- | --- |
| 1. We need to improve the way we deliver care at RIC. | — | .03 | .13*** | .07* | .09* | .08* | .08* | -.04 |
| 1. We have the capability to successfully implement the AbilityLab Model of Care. |  | — | .62*** | .55*** | .49*** | .52*** | .51*** | .45*** |
| 1. Patients will benefit from the change from RIC's current model of rehabilitation to the AbilityLab Model of Care. |  |  | — | .62*** | .51*** | .48*** | .59*** | .51*** |
| 1. I will benefit from the change from RIC's current model of rehabilitation to the AbilityLab Model of Care. |  |  |  | — | .53*** | .52*** | .73*** | .50*** |
| 1. I am prepared to be a part of the AbilityLab Model of Care. |  |  |  |  | — | .69*** | .52*** | .41*** |
| 1. I can implement the AbilityLab Model of Care. |  |  |  |  |  | — | .56*** | .41*** |
| 1. I will experience more self-fulfillment with the AbilityLab Model of Care. |  |  |  |  |  |  | — | .55*** |
| 1. Most of my peers have embraced the AbilityLab Model of Care. |  |  |  |  |  |  |  | — |
| Mean | 3.60 | 3.82 | 3.82 | 3.45 | 3.98 | 3.84 | 3.37 | 3.04 |
| Standard deviation | .88 | .80 | .78 | .88 | .82 | 0.78 | .86 | .91 |
| N | 808 | 804 | 806 | 805 | 804 | 803 | 803 | 806 |
| *Note*. ^*^*p* < .05. ^**^*p* < .01. ^***^*p* < .001 (two-tailed). Correlations are in Spearman’s *r*. | | | | | | | | |

Table S3. *Intercorrelations and descriptive statistics for EBPQ*

| Variable | 1 | 2 | 3 | 4 | 5 |
| --- | --- | --- | --- | --- | --- |
| 1. Seriously questioned whether your default plan of care was the best option. | – | .30*** | .27*** | .12* | .12* |
| 1. Searched the literature to answer a question related to alternative plans of care. |  | – | .73*** | .32*** | .49*** |
| 1. Integrated the evidence you found in the literature with your plan of care. |  |  | – | .50*** | .56*** |
| 1. Evaluated the patient's outcomes to assess if your plan of care was effective. |  |  |  | – | .51*** |
| 1. Shared your practice-based evidence with colleagues. |  |  |  |  | – |
| Mean | 2.97 | 3.21 | 3.34 | 3.80 | 3.31 |
| Standard deviation | .97 | 1.07 | 1.07 | 1.03 | 1.12 |
| N | 410 | 430 | 428 | 434 | 435 |
| *Note*. ^*^*p* < .05. ^**^*p* < .01. ^***^*p* < .001 (two-tailed). Correlations are in Spearman’s *r*. | | | | | |

Table S4. *Intercorrelations and descriptive statistics for EBPAS*

| Variable | 1 | 2 | 3 | 4 | 5 | 6 | 7 |
| --- | --- | --- | --- | --- | --- | --- | --- |
| 1. I like to use new techniques or outcome measures to help my patients. | — | .72*** | .72*** | .48*** | .42*** | .49*** | -.30*** |
| 1. I am eager to use new and different techniques or outcome measures developed by researchers. |  | — | .83*** | .48*** | .42*** | .47*** | -.26*** |
| 1. I would try new techniques or outcome measures even if it were very different from what I am used to doing. |  |  | — | .57*** | .46*** | .53*** | -.26*** |
| 1. it was intuitively appealing? |  |  |  | — | .55*** | .71*** | -.15** |
| 1. it was required by your supervisor? |  |  |  |  | — | .62*** | -.15** |
| 1. it was being used by colleagues who were happy with it? |  |  |  |  |  | — | -.15** |
| 1. I know better than academic researchers how to care for my clients. (reverse scored) |  |  |  |  |  |  | — |
| Mean | 3.90 | 3.72 | 3.80 | 3.85 | 3.74 | 3.88 | 3.00 |
| Standard deviation | .80 | .90 | .84 | .84 | .97 | .89 | 1.31 |
| *N* | 494 | 492 | 495 | 489 | 486 | 484 | 321 |
| *Note*. ^*^*p* < .05. ^**^*p* < .01. ^***^*p* < .001 (two-tailed). Correlations are in Spearman’s *r*. | | | | | | | |
