# Supplementary material for: Pragmatic adaptation of implementation research measures for a novel context and multiple professional roles: a factor analysis study
Source: BMC Health Serv Res. 2020 Mar 30;20:257. doi: 10.1186/s12913-020-05118-4 (PMC7106795; doi:10.1186/s12913-020-05118-4)
Supplement: Supplementary file 2 — Additional file 2: Supplemental File 1. Adapted Versions of the Scales [file 12913_2020_5118_MOESM2_ESM.docx]

# Adapted Implementation Leadership Scale^1^

*Please indicate the extent to which you agree with each statement.*

| **1** | **2** | **3** | **4** | **5** |
| --- | --- | --- | --- | --- |
| **Not at all** | **To a Slight Extent** | **To a Moderate Extent** | **To a Great Extent** | **To a Very Great Extent** |

| 1. RIC's leadership team supports [clinicians' efforts to learn about research/ researchers'   efforts to learn about clinical practice].......................................................................... | 1 | 2 | 3 | 4 | 5 |
| --- | --- | --- | --- | --- | --- |
| 1. RIC's leadership team supports [clinicians' efforts to use research in clinical practice/   researchers' efforts to use clinical practice to drive research development/ employees' efforts to use research to inform their work]................................................................... | 1 | 2 | 3 | 4 | 5 |
| 1. RIC's leadership team recognizes and appreciates employee efforts toward successful implementation of the AbilityLab Model of Care......................................................... | 1 | 2 | 3 | 4 | 5 |
| 1. RIC's leadership team has removed obstacles to implementing the AbilityLab Model   of Care............................................................................................................................ | 1 | 2 | 3 | 4 | 5 |
| 1. My direct supervisor is able to answer my questions about the AbilityLab Model of Care................................................................................................................................ | 1 | 2 | 3 | 4 | 5 |
| 1. My direct supervisor openly addresses problems regarding the implementation of new processes......................................................................................................................... | 1 | 2 | 3 | 4 | 5 |

# Adapted Organizational Change Recipient’s Beliefs Scale^2^

*Please indicate the extent to which you agree with each statement.*

| **1** | **2** | **3** | **4** | **5** | | | | | |  |  |
| --- | --- | --- | --- | --- | --- | --- | --- | --- | --- | --- | --- |
| **Strongly Disagree** | **Disagree** | **Neutral** | **Agree** | **Strongly Agree** | | | | | |  |  |
| 1. We need to improve the way we deliver care at RIC..................................................... | | | | | | 1 | 2 | 3 | 4 | 5 | |
| 1. We have the capability to successfully implement the AbilityLab Model of Care........ | | | | | | 1 | 2 | 3 | 4 | 5 | |
| 1. I can implement the AbilityLab Model of Care............................................................... | | | | | | 1 | 2 | 3 | 4 | 5 | |
| 1. Patients will benefit from the change from RIC's current model of rehabilitation to the AbilityLab Model of Care................................................................................................ | | | | | | 1 | 2 | 3 | 4 | 5 | |
| 1. I will benefit from the change from RIC's current model of rehabilitation to the AbilityLab Model of Care................................................................................................ | | | | | | 1 | 2 | 3 | 4 | 5 | |
| 1. I am prepared to be a part of the AbilityLab Model of Care............................................ | | | | | | 1 | 2 | 3 | 4 | 5 | |

| 1. I will experience more self-fulfillment with the AbilityLab Model of Care.................... | 1 | 2 | 3 | 4 | 5 |
| --- | --- | --- | --- | --- | --- |
| 1. Most of my peers have embraced the AbilityLab Model of Care………….................... | 1 | 2 | 3 | 4 | 5 |

# Adapted Evidence-Based Practice Questionnaire^3^ — Practice Subscale

*Please indicate the extent to which you agree with each statement.*

| 1 | 2 | 3 | 4 |  |  | 5 6 | | | | | | | | |
| --- | --- | --- | --- | --- | --- | --- | --- | --- | --- | --- | --- | --- | --- | --- |
| Never | Rarely | Occasionally | Often |  |  | Very Often N/A | | | | | | | | |
| 1. Seriously questioned whether your default plan of care was the best option.................... | | | | | | | 1 | 2 | 3 | 4 | 5 | 6 |  |  |
| 1. Searched the literature to answer a question related to alternative plans of care............... | | | | | | | 1 | 2 | 3 | 4 | 5 | 6 |  |  |
| 1. Integrated the evidence you found in the literature with your plan of care....................... | | | | | | | 1 | 2 | 3 | 4 | 5 | 6 |  |  |
| 1. Evaluated the patient's outcomes to assess if your plan of care was effective.................. | | | | | | | 1 | 2 | 3 | 4 | 5 | 6 |  |  |
| 1. Shared your practice-based evidence with colleagues...................................................... | | | | | | | 1 | 2 | 3 | 4 | 5 | 6 |  |  |

# Adapted Evidence-Based Practice Attitudes Scale^4^ — Openness Subscale

*Please indicate the extent to which you agree with each statement.*

| 1 | 2 | 3 | 4 | 5 | | | | | |  |
| --- | --- | --- | --- | --- | --- | --- | --- | --- | --- | --- |
| Not at All | To a Slight Extent | To a Moderate Extent | To a Great Extent | To a Very Great Extent | | | | | |  |
| 1. I like to use new techniques or outcome measures to help my patients.......................... | | | | | 1 | 2 | 3 | 4 | 5 | |
| 1. I am eager to use new and different techniques or outcome measures developed by researchers/ I would be eager to do research in a new area if a clinician thought it was important.......................................................................................................................... | | | | | 1 | 2 | 3 | 4 | 5 | |
| 1. I would try new techniques or outcome measures even if it were very different from what I am used to doing................................................................................................... | | | | | 1 | 2 | 3 | 4 | 5 | |

# Adapted Evidence-Based Practice Attitudes Scale^4^ — new subscale comprised of other questions

*Please indicate the extent to which you agree with each statement.*

| **1** | **2** | **3** | **4** | **5** | | | | | |  |  |
| --- | --- | --- | --- | --- | --- | --- | --- | --- | --- | --- | --- |
| **Not at All** | **To a Slight Extent** | **To a Moderate Extent** | **To a Great Extent** | **To a Very Great Extent** | | | | | |  |  |
| 1. It was intuitively appealing?............................................................................................ | | | | | | 1 | 2 | 3 | 4 | 5 | |
| 1. It was required by your supervisor?................................................................................. | | | | | | 1 | 2 | 3 | 4 | 5 | |
| 1. It was being used by colleagues who were happy with it?.............................................. | | | | | | 1 | 2 | 3 | 4 | 5 | |
| 1. I know better than academic researchers how to care for my patients/ I know better than clinicians how new techniques or measures in my area of research could improve patient care…………………………………................................................................... | | | | | | 1 | 2 | 3 | 4 | 5 | |

**References to Original Versions of the Scales/Questionnaires**

1. Aarons GA, Ehrhart MG, Farahnak LR. The Implementation Leadership Scale (ILS): development of a brief measure of unit level implementation leadership. *Implement Sci.* 2014;9(1):45.

2. Armenakis AA, Bernerth JB, Pitts JP, Walker HJ. Organizational Change Recipients Beliefs Scale: Development of an Assessmetn Instrument. *The Journal of Applied Behavioral Science.* 2007;42:481-505.

3. Upton D, Upton P. Development of an evidence-based practice questionnaire for nurses. *J Adv Nurs.* 2006;53(4):454-458.

4. Aarons GA. Mental health provider attitudes toward adoption of evidence-based practice: the Evidence-Based Practice Attitude Scale (EBPAS). *Ment Health Serv Res.* 2004;6(2):61-74.
